# Punctate Midline Myelotomy for Chronic, Intractable, Non-malignant Visceral Pain: A Case Report

**DOI:** 10.7759/cureus.5028

**Published:** 2019-06-28

**Authors:** Zaid Aljuboori, Tyler Ball, Haring J Nauta

**Affiliations:** 1 Neurosurgery, University of Louisville School of Medicine, Louisville, USA

**Keywords:** punctate midline myelotomy, limited midline myelotomy, functional chronic visceral pain, visceral pain, chronic pain, abdominal pain, interventional pain, myelotomy, pain control, postsynaptic dorsal column pathway

## Abstract

Punctate midline myelotomy (PMM) has a strong anatomic and functional basis for its role in the treatment of visceral pain. The procedure derived from advances in the understanding of the postsynaptic dorsal column (PSDC) pathway and the converging laboratory and clinical evidence that this spinal cord pathway plays a dominant role in conveying visceral pain to higher levels of the nervous system. The result of PMM is a concise, effective interruption of the PSDC pathway with minimal to no side effects. While considerable evidence now documents that PMM has good efficacy and safety in treating malignant visceral pain, there is little experience describing its application to chronic severe refractory visceral pain of benign origin.

We present the case of a patient with a 13-year history of severe non-malignant chronic abdominal visceral pain who obtained complete pain relief from a PMM at the T7 level. Intraoperative somatosensory evoked potential (SSEP) monitoring did not show changes after making the PMM lesion. As of six-months postoperative follow-up, the benefit shows no sign of fading, all pain medications have been discontinued, and there has been no impairment of motor function, bowel or bladder function, sexual function, gait or station. Upon detailed questioning, the patient endorsed only mild subjective reduced sensation of the inner aspects of her feet that was not bothersome to her. On detailed testing, position sense was preserved throughout; the Romberg test was negative, and the only finding was reduced vibratory sensation over the great toe pads. We cautiously suggest that the PMM operation may allow relief from severe, intractable, benign visceral pain syndromes for which effective treatments are otherwise elusive. The procedure warrants further study for such conditions.

## Introduction

Functional gastrointestinal disorders, inflammatory bowel disease, gastric paresis, interstitial cystitis, and chronic pelvic pain in women, are examples of sometimes intractable non-cancerous functional chronic visceral pain (FCVP) syndromes [1-2]. They can be challenging to control by any currently available means. Here we present a patient with chronic intractable visceral pain of non-malignant origin who was successfully treated by mid-thoracic punctate midline myelotomy (PMM). This procedure was developed specifically to interrupt the ascending fibers of the postsynaptic dorsal column (PSDC) pathway by means of a very concise, short transvers cut [3-4] across the midline of the dorsal columns, interrupting only the medial 1 mm of the dorsal columns to each side. The PSDC pathway ascends along the midline of the dorsal columns and is now understood to be the principal pathway by which visceral pain signals are conveyed to the thalamus [5-6]. The lesion produced by PMM is so small and so specific that it does not appear to impair the classically understood functions of the dorsal columns related to position sense, gait or station. Interruption of the PSDC pathway either by the small transverse cut of PMM [3-4,7], or by sagittal cuts in variants of commissural myelotomy [6], has proven effective for relief of cancer-related pelvic visceral pain and other visceral pain [8] with minimal or no additional deficits. However, any potential role of PMM in managing non-malignant pain remains to be defined.

## Case presentation

The patient is a 26-year-old woman with a 13-year history of chronic abdominal pain that started after an episode of presumed "food poisoning." The pain typically lasted 5-6 hours per day, was triggered by eating or bowel movements, and was often severe enough to induce vomiting. It was causing significant interference with her daily activities, both from the distraction of the pain itself and from the side effects associated with pain medication. The patient found an article about PMM performed on a series of six cancer patients and requested evaluation and consideration for the operation. At the time of our first evaluation, the patient had already undergone multiple colonoscopies with no abnormalities identified other than some nonspecific inflammation. She had also undergone endoscopy with biopsy of her gastrointestinal tract, in addition to a pill camera study. These revealed only small intestinal overgrowth. She even underwent a diagnostic laparotomy that did not show any abnormalities. She carried a diagnosis of severe irritable bowel syndrome with constipation (IBS-C) with visceral hypersensitivity syndrome. Regarding prior therapies, she had tried a multitude of diets without relief, and had even gone on total parenteral nutrition (TPN) for months for bowel rest with no resolution of her symptoms. Notably, her insurance company had denied other surgical options including a spinal cord stimulator and an intrathecal morphine pump. On exam, she was neurologically intact other than diffusely reduced deep tendon reflexes, more so in the upper extremities. Given the nature and severity of the pain, the extensive and unrevealing prior workup and therapeutic strategies, and the patient’s wishes, we offered a PMM as a treatment option for her clearly visceral origin pain. We discussed the risks in detail, as well as the uncertainty given the paucity of clinical experience with PMM in non-malignant pain. Despite these cautions, the patient and family wished to proceed with surgery.

**Figure 1:**
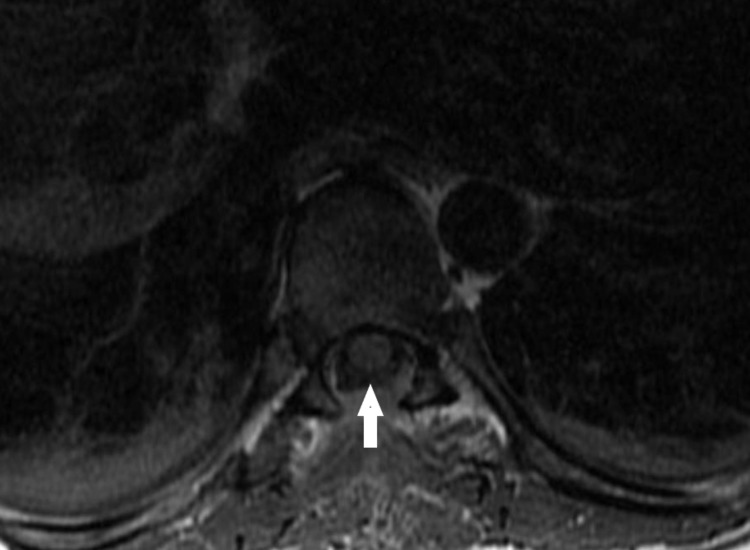
Post-operative axial T1 magnetic resonance imaging (MRI) showing the transverse dimension of the tiny dorsal column lesion in the midline at the T7 level of the thoracic spinal cord The linear hyperintensity above the arrow measures approximately 2 x 5 mm.

The patient underwent an uneventful PMM via a T7 laminectomy. The technique used was that of a simple transverse crush injury to the PSDC system as first described by Nauta and colleagues in 2000 [4]. Figure 1 depicts the post-operative magnetic resonance imaging (MRI) of the lesion.

Intraoperatively, she had no decrement in her somatosensory evoked potential (SSEP) monitoring. Anticipating difficulty with post-operative pain control from the patient’s tolerance to opioids, liposomal bupivacaine (EXPAREL®) was injected intraoperatively into the paraspinal muscles and wound edges. Over the first two post-operative days, she was uncertain of any benefit. Then on post-operative day three, she had a bowel movement which was the first in many years without associated severe pain. By two-week follow-up, the patient had experienced no episodes of abdominal pain since the procedure and had weaned her pain medication from 2 mg of oral hydromorphone every 4-5 hours pre-operatively to 2 mg every 12 hours for incisional pain. On exam, she was motor intact with preserved light touch and proprioception in her lower extremities. The only detectable deficit was diminished sensation to vibration on her great toe pads bilaterally. Her gait was normal and Romberg testing was negative. Regarding sensory changes, she reported only mild numbness of the vulva but not of the vagina and some numbness on the insides of her feet. By her eleven-week follow-up, she was pain free off all opioid medication. She was able to eat whatever food she liked and was having normal pain free bowel movements. Also, the previous numbness/tingling in her vulva had resolved, and she had regained full sensation. She was having no balance difficulties, nor was she having bowel or bladder function issues. Moreover, she was again able to exercise and succeeded in losing over 10 pounds since surgery. Her only perceptible abnormality was some tingling in her toes bilaterally and around her incision on her back. Notably, her sexual function was normal, and she was able to achieve orgasm as pre-operatively. Overall, the patient was extremely pleased with the results of the operation.

## Discussion

While substantial progress has been made in the assessment and treatment of chronic somatic pain, there has been little success in the management of FCVP [1]. FCVP and visceral hyperalgesia may include both central and peripheral nervous system components and may be maintained by either [9]. Chronic episodic pain is the most common complaint with functional gastrointestinal disorders, as is heightened perception of gastrointestinal sensation (visceral hypersensitivity) [10]. Patients often undergo extensive diagnostic workups which may themselves carry significant risks, and they often try a multitude of medical and surgical treatments with no or only incomplete relief.

Chronic visceral pain, both cancer and non-cancer related, has a multitude of negative effects on afflicted patients, including psychological distress, inability to work, financial burden, opiate addiction, and others [1,11]. Studies have shown that patients with chronic visceral pain are more likely to develop anxiety and depression [11]. Also, opioids have been increasingly used in the management of chronic non-cancer related visceral pain given the lack of effective alternatives [1]. Birnbaum and colleagues demonstrated that patients who abused opioids incurred a significantly higher total direct health care costs per patient when compared to demographically matched controls [12]. Additionally, chronic opioid use put the patients at risk for opioid overdose. A study by the Center for Disease Control showed that 41% of drug overdose deaths in 2008 resulted from opioid overdose [13].

Given these statistics, as well as the detrimental effects of chronic pain, we aim to apply knowledge of a well described anatomic pathway to a new population in need of treatment alternatives. Dorsal column midline myelotomy, and the less destructive PMM, have been proven effective in providing sustained relief of cancer-related visceral pain [3-4,7]. They have a higher success rate in comparison to anterolateral cordotomy or commissural myelotomy for this type of pain [14]. There is also a better safety profile. Visceral abdominal pain transmission is typically bilateral in origin, which means that bilateral anterolateral cordotomies would be required, but that procedure is known to have severe negative consequences such as central respiratory failure, bladder, and rectal dysfunction, and lower extremity weakness [14-16]. Alternatively, a commissural myelotomy at multiple spine segments can be performed, but again that procedure carries significant risks including loss of pain sensation, weakness, bladder dysfunction, and imbalance [17-18].

There is currently understandable caution accepting the idea of an ablative procedure for pain of a “benign” cause. PMM so far is well characterized only for palliation of malignant origin pain. The successful treatment of cancer patients with limited life expectancy still leaves unanswered questions concerning the longer-term durability of the benefit and possible risks of late delayed pain syndromes. However, some clues may be gleaned from the limited reports of older procedures which probably exerted their benefit by interrupting the PSDC pathway, although inadvertently. These older procedures were made with a cut in the midsagittal plane with the intended goal of interrupting the bilateral fibers of the spinothalamic pathway crossing midline near the segmental level of entry. The extensive pattern of pain relief observed by Sourek [8] and Hitchcock [19] following even short midline lesions led each of these authors to propose that some ascending pathway had been interrupted. A few patients in Sourek’s series had longer follow up, up to 36 months. They experienced no recurrence of their pain, while their additional analgesic girdle-shaped area had normalized by six months after surgery [8].

The experience with anterolateral cordotomy has led to an understandable hesitation to apply ablative techniques to patients with longer life expectancies since cordotomy patients often had return of their pain over a period of months. Moreover, in a small percentage of patients, the original pain was replaced by a “post cordotomy dysesthesia,” a condition for which no effective treatment could be offered. However, there is no reason to assume that these shortcomings of anterolateral cordotomy would necessarily apply to interventions on the PSDC pathway. However, until there is reassurance from more experience, the PMM procedure should probably only be used when medical options have been exhausted, or when the side effects of medication are intolerable. Also, other options, such as a spinal cord stimulator or intrathecal morphine pump, should at least be considered before proceeding with PMM for benign pain. When weighing the risks and benefits of the procedure, it is also fair to consider that treatment of pain with long term high-dose opioid therapy may not be very effective and puts patients at significant risk for addiction, tolerance, and a variety of negative side effects [20]. Additionally, long term opioid use results in a financial burden on both the patient and the healthcare system [12]. Similarly, the use of spinal cord stimulation or an intrathecal pain pump has many potential drawbacks, including infection, the need for long term maintenance, and complications of device failure, catheter tip granuloma, and spinal cord injury. Also, the inherent device costs can preclude their use in many parts of the world. Thus, the impetus remains strong to find a better solution for patients with benign intractable visceral pain syndromes.

Because of the success our patient had with this operation, as well as anecdotal reports of long-term pain relief without serious neurologic sequelae based on experiences with PSDC interruption in cancer patients and applicable reports in the older literature, we believe that the specific interruption of the PSDC pathway by PMM should be considered in patients with intractable pain of visceral origin. As we gather more experience applying PMM to different chronic intractable pain conditions, we are likely to learn which characteristics predict a robust response. For example, in the case reported here, it may be important to note that her pain was convincingly visceral in origin, and was intermittently severe in a way that may have limited any progress towards centralization thought to occur with constant chronic pain. 

## Conclusions

Many chronic visceral pain syndromes are intractable without known effective treatments. They can be sources of severe physical, psychological, and financial burden for patients. In patients who have failed medical management, or have intolerable side effects from medical management, surgical interruption of the PSDC pathway by PMM may provide dramatic and sustained pain relief with minimal to no long-term side effects. This method warrants further consideration and study for these conditions.
